# The Editor’s Choice for Issue 2, Volume 7

**DOI:** 10.3390/ijns7030061

**Published:** 2021-09-18

**Authors:** Can Ficicioglu

**Affiliations:** Division of Human Genetics/Metabolism, Children’s Hospital of Philadelphia, Perelman School of Medicine at the University of Pennsylvania, Philadelphia, PA 19104, USA; ficicioglu@email.chop.edu

Dear readers: I am proud to say that we are continuing to publish many important papers on newborn screening in *IJNS*, and the papers published in this issue clearly support my statement. Please allow me to highlight two papers published by Basheeruddin et al. [1] and Corre et al. [2] on newborn screening for Krabbe disease (KD) in this issue. Much more information is needed regarding this rare disease to inform discussion among researchers, clinicians and public advocates about whether it should be included as a part of the federal RUSP in the United States.

In the United States, the expansion of newborn screening panels is guided by the Advisory Committee on Heritable Disorders in Newborns and Children and the Secretary of Health and Human Services. Nominated conditions are reviewed and vetted by this group and then approved by the Secretary of Health. Once it is approved, that condition becomes part of the federal Recommended Uniform Screening Panel (RUSP). Many states observe this recommendation and include recommended disorders in their state NBS panel. In 2009, by an eight-to-seven vote, it was not recommended that KD should be added to the RUSP [3]. The advisory board offered several explanations for its closely contested decision. It called for: (1) a better definition of disease types; (2) improved screening methods; (3) more data on the efficacy of existing treatment options. Over the years, patient advocacy has played a large role in the addition of KD NBS to state panels. New York State implemented newborn screening for KD in 2006. Newborn screening (NBS) for KD is currently practiced in nine U.S. states (Indiana, Kentucky, Missouri, New Jersey, New York, Ohio, Illinois, Pennsylvania, and Tennessee). There is continued discussion of whether it should be implemented in additional states.

The early diagnosis and treatment of KD, especially early infantile forms, is crucial, and the only way is to screen this disease in the first days of life. Newborn screening is performed by measuring galactocerebrosidase (GALC) enzyme activity in dried blood spots (DBSs). GALC DNA sequence analysis is available as a second-tier test. It is helpful to exclude cases which exhibit low enzyme activity due to pseudodeficiency alleles. However, such analysis could be inconclusive if cases have variants of unknown significance (VUS) that partially reduce the activity of GALC.

A biomarker test measuring the levels of psychosine has been developed to increase the sensitivity of screening for KD [4]. Disease-specific reference ranges of psychosine have been established for infantile-onset KD (IKD) and for late-onset KD. Experts agree that performing biomarker tests as part of screening is important and will strengthen the case for KD’s inclusion in the RUSP. We have two published papers on KD newborn screening and psychosine in this issue.

The first paper by Basheeruddin et al. [1] presents three years of Krabbe NBS experience in Illinois and discusses the results of analyzing psychosine as a second-tier test. The Illinois state NBS program performs GALC enzyme test as a first-tier assay and adds genetic tests with analyses of psychosine levels when newborns have low GALC enzyme levels. Newborns with pseudodeficiency alleles were not followed up, and all had psychosine levels of less than 2 nM. A follow-up was not recommended for newborns exhibiting VUS or being carriers (a single pathogenic mutation or one copy of the 30 kb deletion). The assays showed that 5 of 35 newborns presented with VUS and 7 of 67 carriers had psychosine levels between 2 and 3 nM. Two newborns were diagnosed with infantile forms of Krabbe disease, with psychosine levels of 10 and 35 nm, respectively.

Six newborns had low levels of GALC activity detected. Some had two pathogenic mutations and were classified as late-onset; those with one pathogenic and one VUS mutations were defined as probable late-onset. All six had mildly elevated psychosine levels, between 2 and 6 nM. They were diagnosed as late-onset or probable late-onset and followed up in clinics.

Basheeruddin et al. decided which patient needed following up based on genetic tests and psychosine levels. Assuming that variants are less likely to be disease-causing, they did not follow up cases with two VUS mutations and slightly elevated psychosine levels.

The second paper, reported by Corre et al. [2], makes a significant contribution to adjusting the cut-off value of psychosine, and establishing follow-up algorithms. The authors present a case detected through the NY NBS program. The infant exhibited low enzyme activity, with two likely pathogenic mutations but normal psychosine levels. The value of psychosine was 1.6 nmol/L at the second day of life, and the repeat was 2.6 nmol/L (cutoff 3 nmol/L) at 2 weeks of age. The parents reported irritability, frequent crying, and poor sleep quality at 3 months of age. The psychosine marker was repeated at 7 months of age and decreased to 0.8 nmol/L. This patient had worsening neurological symptoms, although the repeated psychosine values ranged from 1 and 2 nmol/L. The MRI findings were not completely typical of early-onset infantile Krabbe disease (EIKD), but whole-exome sequencing did not indicate any other possible diagnosis. This is a puzzling case because all reported cases with EIKD have elevated psychosine levels, except for one 2-month-old patient where the condition was detected based on family history [5].

I believe that there are three important take-home messages from these two papers: (1) psychosine as a second-tier test is important and can increase the precision of screening for Krabbe disease; (2) it is essential to fully take into account a combination of all genetic tests and biomarkers when deciding how to monitor these cases. Biochemical tests are invaluable when interpreted in concert with careful attention to a patient’s symptoms; (3) clinicians must continue to share case histories so that we can learn from each another as we seek to establish best practices for our patients identified through NBS.
